# Differences in the Acute Effects of Aerobic and Resistance Exercise in Subjects with Type 2 Diabetes: Results from the RAED2 Randomized Trial

**DOI:** 10.1371/journal.pone.0049937

**Published:** 2012-12-05

**Authors:** Elisabetta Bacchi, Carlo Negri, Maddalena Trombetta, Maria Elisabetta Zanolin, Massimo Lanza, Enzo Bonora, Paolo Moghetti

**Affiliations:** 1 Section of Endocrinology, Diabetes and Metabolism, Department of Medicine, University and Azienda Ospedaliera Universitaria Integrata of Verona, Verona, Italy; 2 Section of Epidemiology and Medical Statistics, Department of Public Health and Community Medicine, University of Verona, Verona, Italy; 3 Section of Motor Sciences, Department of Neurological, Neuropsychological, Morphological and Movement Sciences, University of Verona, Verona, Italy; University of Bath, United Kingdom

## Abstract

**Objective:**

Both aerobic (AER) and resistance (RES) training, if maintained over a period of several months, reduce HbA1c levels in type 2 diabetes subjects. However, it is still unknown whether the short-term effects of these types of exercise on blood glucose are similar. Our objective was to assess whether there may be a difference in acute blood glucose changes after a single bout of AER or RES exercise.

**Study Design:**

Twenty-five patients participating in the RAED2 Study, a RCT comparing AER and RES training in diabetic subjects, were submitted to continuous glucose monitoring during a 60-min exercise session and over the following 47 h. These measurements were performed after 10.9+0.4 weeks of training. Glucose concentration areas under the curve (AUC) during exercise, the subsequent night, and the 24-h period following exercise, as well as the corresponding periods of the non-exercise day, were assessed. Moreover, the low (LBGI) and high (HBGI) blood glucose indices, which summarize the duration and extent of hypoglycaemia or hyperglycaemia, respectively, were measured.

**Results:**

AER and RES training similarly reduced HbA1c. Forty-eight hour glucose AUC was similar in both groups. However, a comparison of glucose AUC during the 60-min exercise period and the corresponding period of the non-exercise day showed that glucose levels were lower during exercise in the AER but not in the RES group (time-by-group interaction p = 0.04). Similar differences were observed in the nocturnal periods (time-by-group interaction p = 0.02). Accordingly, nocturnal LBGI was higher in the exercise day than in the non-exercise day in the AER (p = 0.012) but not in the RES group (p = 0.62).

**Conclusions:**

Although AER and RES training have similar long-term metabolic effects in diabetic subjects, the acute effects of single bouts of these exercise types differ, with a potential increase in late-onset hypoglycaemia risk after AER exercise.

**Trial registration:**

ClinicalTrials.gov NCT01182948

## Introduction

Current guidelines for exercise in subjects with type 2 diabetes recommend performing, whenever possible, both aerobic and resistance exercise training [1]. Interestingly, some recent comparative studies showed that these exercise modalities, if maintained over a period of several months, are similarly effective in reducing HbA1c levels in diabetic patients [2]–[4]. However, it is unknown whether the acute blood glucose changes induced by each single session of aerobic or resistance exercise are also similar. As a consequence, it is still unclear whether medication dosage and/or dietary carbohydrate adjustments to exercise should differ according to the scheduled type of physical activity.

A single bout of exercise increases skeletal muscle glucose uptake. Nevertheless, plasma glucose is normally maintained constant by increased hepatic glucose production and mobilization of alternate fuels [5]. In subjects with type 2 diabetes performing moderate aerobic physical activity, glucose uptake generally rises more than hepatic glucose production, and therefore blood glucose tends to fall [6]. Muscle glucose uptake remains high in the post-exercise period, with the contraction-mediated pathway persisting for several hours and insulin-mediated uptake for longer [7]. This phenomenon is usually beneficial in these patients and, in the absence of pharmacological interference, concurrent reduction of insulin levels avoids the risk of hypoglycaemia. However, the synergistic effects of exercise and antidiabetic medications may provoke a more pronounced reduction of glucose concentrations [8]. This interaction may still be helpful in improving the metabolic control. Nevertheless, it increases the risk of hypoglycaemia, which may occur either during or several hours after the exercise.

In clinical practice, assessment of short-term changes in glucose concentrations is primarily based on self-monitoring of blood glucose. However, most hypoglycaemic episodes may be missed [9]–[10], particularly during the night. A recently introduced tool to assess blood glucose changes, available during exercise and free-living, is the continuous glucose monitoring system (CGMS), which can record blood glucose concentrations for several days.

In the present study, using CGMS, we have compared the effects on blood glucose of a single bout of aerobic exercise or resistance exercise in a group of type 2 diabetic patients included in a regular training programme. Our hypothesis is that aerobic exercise and resistance exercise, despite similar effects on long-term metabolic control in these subjects, may differently affect glucose concentrations during and for several hours after the exercise.

## Materials and Methods

### Ethics statement

The study was approved by the Ethical Committee of the Azienda Ospedaliera Universitaria Integrata of Verona, Italy, and written informed consent was obtained from all participants. All investigations were conducted according to the principles expressed in the Declaration of Helsinki.

The protocol for this trial and supporting CONSORT checklist are available as supporting information; see Checklist S1 and Protocol S1.

### Design

This is a sub-project of the RAED2 study, a randomized controlled trial aimed at comparing the metabolic effects of aerobic and resistance training in type 2 diabetes subjects. In this trial, 60-min exercise sessions were scheduled 3 times weekly, on alternate days, for 4 months.

In this sub-project, blood glucose was continuously monitored by CGMS over 48-h, starting with an exercise session.

To adequately assess the effects of the two exercise modalities, the CGMS sensor was implanted in all subjects after at least two months of training, i.e. when scheduled exercise volumes had been reached and maintained for several weeks.

### Subjects

**Figure 1:**
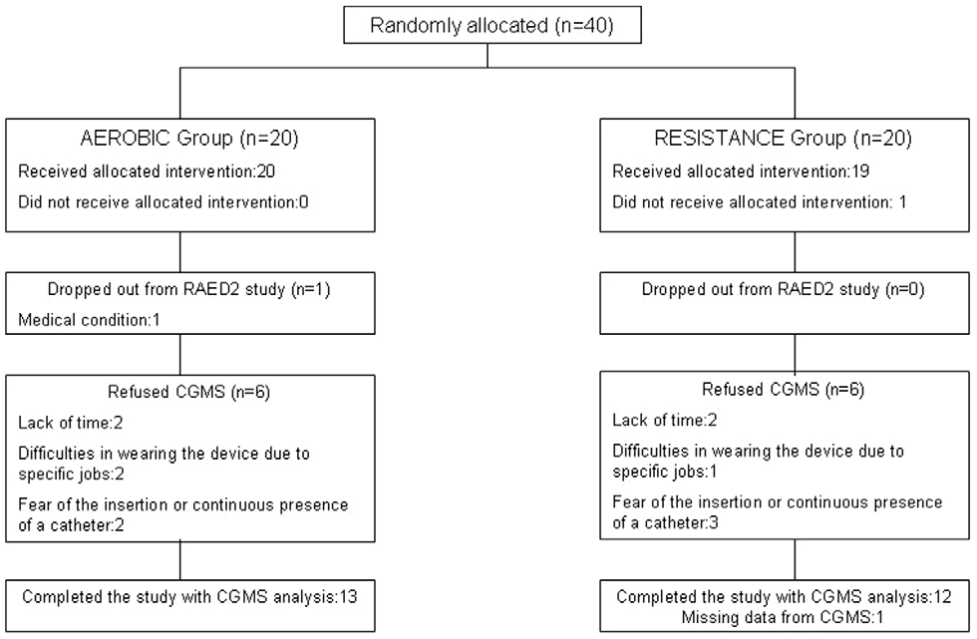
Study flow diagram.

Inclusion and exclusion criteria of the study, sample size calculation and randomization schedule have been reported in detail elsewhere [4]. The only allowed diabetes medications were oral hypoglycaemic agents. Of the 40 eligible patients enrolled in the RAED2 study, 38 completed the trial and 26 of them agreed to participate in this sub-project and had the CGMS implanted. In one of these subjects the CGMS failed to record the data. Therefore, glucose measurements were collected in 25 subjects, 13 from the aerobic and 12 from the resistance group (Fig. 1).

### Training protocols

The aerobic group exercised 3 times a week on different cardiovascular equipment (treadmill, cyclette and elliptical). During the initial phase (weeks 1–2), participants exercised for 30–40 min at 40–60% of the reserve heart rate. Thereafter, training duration gradually increased up to 60 min per session and, subsequently, intensity gradually reached 60–65% of the reserve heart rate. The target training intensity was achieved after 5 weeks in all subjects of the AER group. Heart rate monitors (Polar S810i; Polar Electro, Kempele, Finland) were used to standardize exercise intensity.

The resistance group performed 9 different exercises in 3 weekly sessions, by using weight machines and free weights. During the learning phase (weeks 1–2) subjects performed 3 series of 10–12 repetitions at 30–50% 1RM. Subsequently, the amount of weight lifted increased progressively. All participants were asked to perform each repetition in a slow, controlled manner, with a rest of 60 s between sets. The training workload was increased after subjects had successfully achieved 12 repetitions with an appropriate technique. The target intensity of the training programme (10–12 repetitions at 70–80%1RM) was reached after 6 weeks in all subjects of the RES group.

All training sessions were supervised by exercise specialists. In this sub-project, the exercise sessions started for all subjects at 6:30 p.m.

### Continuous glucose monitoring system

The CGMS measures interstitial glucose concentrations via the glucose oxidase reaction, with the enzyme immobilized on a subcutaneously inserted electrode. The CGMS used was the Guardian REAL-Time (Medtronic Minimed, Inc., Northridge, CA, USA), which consists of a sensor-transmitter implanted into the abdominal subcutaneous tissue and a reader-monitor with wireless radio frequency sensor. This system was previously validated [11].

For this protocol, the patient checked into to the Diabetes Clinic of Verona Hospital in the morning, after a 12-h overnight fast. The CGMS was inserted at 8:30–9:30 am by an experienced physician, following the manufacturer's instructions. The sensor's display and the patient's watch were both synchronized with the clinical trial unit clock. Self-measured blood glucose was used to calibrate the instrument and the first calibration was performed 2 h after initialization. If no abnormalities were observed, the patients were subsequently discharged. Thereafter, the patients were asked to maintain their normal daily activities and diet. During registration, the participants self-measured blood glucose at least four times per day and values were used to calibrate the CGMS, as required by the device. However, subjects were blinded to CGMS measurements. After about 60 h the sensors were removed and the data collected.

### CGMS data

We analysed glucose concentration data recorded in a 48-h period, starting at 6:30 pm on the day in which the CGMS sensor was implanted, corresponding to the beginning of a 60-min exercise session.

**Figure 2:**
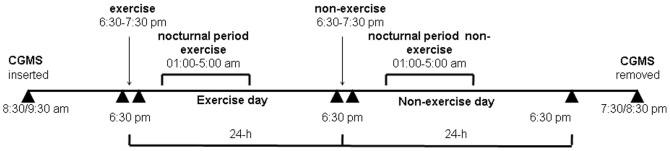
Schematic overview of the study design. The CGMS sensor was inserted at 8:30–9:30 am. Glucose concentrations were recorded over a 48-h period, starting at 6:30 pm of the same day, corresponding to the beginning of the 60-min exercise session. Several time periods were separately analyzed: the exercise period (6:30–7:30 pm), the subsequent nocturnal period (1:00 am–5:00 am) and the 24-h period following the beginning of the exercise session (exercise day), as well as the corresponding time periods of the following (non-exercise) day. The CGMS was removed at 7:30–8:30 pm of the non-exercise day. Meal times were between 6:30–8:30 am for breakfast, 12:30 am to 2:00 pm for lunch, 3:30–4:30 pm for a snack and 8:00–9:30 pm for dinner.

Analyses were carried out over the entire 48-h period and in several selected time periods. These comprised the 6:30–7:30 pm exercise period, the subsequent nocturnal period, and the 24-h period following the beginning of the exercise session (exercise day), as well as the corresponding time periods of the following (non-exercise) day. The nocturnal period was defined as the time period in which all subjects included in the study were asleep, corresponding to the interval 1:00–5:00 am. An overview of the study is provided in Figure 2.

Hypoglycaemias were classified following the ADA Workshop on Hypoglycaemia definitions (severe hypoglycaemia, documented symptomatic hypoglycaemia, or asymptomatic hypoglycaemia) [12]. In particular, we defined an asymptomatic hypoglycaemia as an event not accompanied by typical symptoms of hypoglycaemia but with a measured plasma glucose concentration ≤70 mg/dl; symptomatic hypoglycaemia as an event during which typical symptoms of hypoglycaemia are accompanied by a measured plasma glucose concentration ≤70 mg/dl, and severe hypoglycaemia as an event requiring assistance of another person to actively administer carbohydrate, glucagons, or other resuscitative actions.

The low blood glucose index (LBGI) and the high blood glucose index (HBGI) were calculated by using the formulas proposed by Kovatchev et al, as adapted for the frequently sampled CGMS output [13]. These indices summarize in a single number the percentage of low (<70 mg/dL) or high (>180 mg/dL) glucose readings, respectively, and their magnitude. LBGI provides a reliable assessment of severe hypoglycaemia risk [14].

### Other measurements

Weight was recorded on an electronic scale (Tanita BWB-800, MA, USA), height was measured with a Harpenden stadiometer (Holtain Ltd., Crymych Pembs, UK), and BMI was calculated as weight (kg)/height^2^ (m).

HbA1c was measured by a Diabetes Control and Complications Trial (DCCT)-aligned method, with an automated high performance liquid chromatography analyzer (Bio-Rad Diamat, Milan, Italy).

VO2peak was measured during a cycle ergometer incremental stress test by breath-by-breath analysis of oxygen consumption and carbon dioxide production. After a warm-up load of 30 W for 3 min, 10-W increments were applied each minute up to voluntary exhaustion.

### Diet

All participants in the RAED2 study received preliminary healthy dietary instructions, as previously described [4]. Moreover, subjects included in this sub-project were asked to maintain their diet and medications stable over the 48-h study period. The food intake was divided into breakfast, lunch, dinner and a snack. Meal times were between 5:30–8:30 am for breakfast, 12:30 pm to 2:00 pm for lunch, 3:30–4:30 pm for a snack and 8:00–9:30 pm for dinner, and remained constant for each subject in the exercise and non-exercise day. Subjects were also asked to fill in a 2-day food recall questionnaire. The questionnaires were subsequently analyzed, by a trained dietician, with MetaDieta software Vers.3.1, (Me.Te.Da, AP, Italy).

### Statistical analysis

Analyses were carried out using STATA version 11 (StataCorp, College Station, Texas, USA). Data are shown as mean and Standard Error (SE), or median and interquartile range (IQR), as appropriate. Normality of the distribution of the studied variables was assessed by the Shapiro-Wilk test. Skewed variables were log transformed before analysis.

The baseline characteristics of the two training groups were compared by Student t-test or Fisher exact test, as appropriate. Median attendance to exercise sessions between the two groups was compared by Wilcoxon rank-sum test.

The area under the curve (AUC) of glucose levels was calculated over the entire 48-h registration period and in selected periods of the exercise and non-exercise days. Repeated measures ANOVA was used to compare differences between the exercise and non-exercise day in the two groups, with the AUCs (or the AUC logarithms) as the dependent variables and time, study group, and time-by-group interaction as independent variables. In this analysis, particular attention was given to the interaction term as its significance meant a different trend in the dependent variable in the two groups; when this was true, separate Student t-test for paired data were performed in both groups.

As LBGI and HBGI could not be normalized, non parametric tests were used to compare the differences of these variables between the exercise and non-exercise day (Wilcoxon matched-pair test) and between groups (Wilcoxon rank-sum test). The Bonferroni correction was applied in these analyses to adjust for multiple comparisons.

Multiple regression analysis was carried out to assess whether there were additional variables independently associated with glucose changes. In this analysis gender, medication category (insulin sensitizers vs secretagogues), age, and baseline glucose levels were assessed as predictors of outcomes.

Tests with p<0.05 were considered statistically significant.

## Results

Subjects of the aerobic and resistance training groups participating in the sub-project had similar baseline characteristics (Table 1). These features were also similar to those of the whole cohort of subjects enrolled in the RAED 2 study (data not shown).

**Table 1:** Participants Baseline Characteristics.

	Aerobic Group (N = 13)	Resistance Group (N = 12)	P value
**Men/Women** (n)	9/4	7/5	0.69
**Age** (years)	57.0±2.1	56.1±2.4	0.76
**BMI** (kg/m^2^)	29.5±1.2	31.6±1.2	0.22
**Duration of Diabetes** (years)	10.7±1.8	9.2±2.7	0.66
**HbA1c** (%)	7.3±0.2	7.5±0.2	0.54
**Antidiabetic Therapy** (n)			0.64
**Diet alone**	1	0	
**Metformin**	7	7	
**Thiazolidinediones**	2	0	
**Sulfonylureas**	0	1	
**Meglitinides**	0	1	
**Metformin and Sulfonylureas**	3	2	
**Metformin and Meglitinides**	0	1	
**VO_2peak_** (mL·kg^−1^·min^−1^)	25.5±0.9	24.8±1.6	0.71
**Overall physical activity** (MET min per week)	329±63	255±64	0.42
**Caloric Intake** (Kcal/day)	1468±122	1373±68	0.31

Values are mean±SE unless otherwise specified.

At the time the CGMS was implanted, after 10.9±0.4 weeks of training, median attendance to supervised exercise sessions was similar in the aerobic and resistance groups (93.5% vs 90.2% respectively, p = 0.64).

Analysis of the 2-day food recall questionnaire, filled in by participants during the CGMS registration period, showed that calorie intake (1581±191 vs 1504±149 in the AER group, and 1468±60 vs 1482±194 kcal in the RES group, respectively in the exercise and non-exercise days) was similar over the two days and in both groups. Diet composition was also similar. In particular, there were no differences in carbohydrate intake (196±27 vs 194±20 in the AER group, and 183±13 vs 180±36 g in the RES group, respectively in the exercise and non-exercise days).

The glucose concentrations AUC over the entire 48-h CGMS registration did not differ between groups [6253±250 vs 6318±457 (mg/dL) · h, p = 0.90]. Mean glucose levels during this time period were 126±5 vs 134±8 mg/dL, respectively in the AER and RES groups (p = 0.37). Glucose readings at the beginning of the exercise session were also similar in both groups (119±5 vs 135±7 mg/dL, p = 0.45).

Table 2 shows the glucose concentration AUCs in the different time periods assessed during both exercise and non-exercise days, in the two groups. In both groups, the 24-h glucose AUC did not differ between the exercise and non-exercise days, and there were no differences, on both days, between the AER and RES groups.

**Table 2:** Glucose concentration AUCs, and LBGI and HBGI values during the exercise and non-exercise days in the Aerobic and Resistance groups.

	Aerobic group (N = 13)		Resistance group (N = 12)			
	exercise	non-exercise	exercise	non-exercise	P value *Time*	P value *Time-by-group Interaction*
**Glucose AUCs** (mg/dL)·h						
**Session day-time (6:30–7:30)**	**117 (104–125)** a	131 (119–154)	133 (116–164)	140 (125–145)	**-**	**0.04**
**Nocturnal period (01:00–05:00)**	**363 (338–473)** a	519 (367–595)	476 (393–525)	502 (454–540)	**-**	**0.02**
**24-h**	2980 (2589–2509)	3073 (2754–3399)	3063 (2681–3314)	3203 (2826–3700)	0.45	0.34
**LBGI**						
**Nocturnal period (01:00–05:00)**	**1.27 (0–2.92)** b	0 (0–1.55)	0.02 (0–1.64)	0 (0–1.93)		
**24-h**	0.37 (0.07–1.45)	0.18 (0.03–0.77)	0.01 (0–0.96)	0.03 (0–0.99)		
**HBGI**						
**24-h**	2.12 (0.65–3.20)	1.52 (0.57–2.43)	1.68 (0.77–2.11)	1.60 (1.33–6.61)		

Data are median and interquartile range.

P values refer to comparisons between groups by repeated measures ANOVA. Statistically significant figures are in bold type. When a significant time-by-group interaction was found, differences within each group between the exercise and the corresponding non-exercise values were assessed by Student's t test, and statistically significant figures were indicated by symbols. Wilcoxon non parametric test was used to compare LBGI and HBGI values between the exercise and non-exercise days (Wilcoxon matched-pair test) and between groups(Wilcoxon rank-sum test), and statistically significant figures were indicated by symbols.

a p<0.05 vs non-exercise;

b p<0.01 vs non-exercise.

However, comparison of the glucose AUC during the 60-min exercise session and the corresponding 60-min period of the non-exercise day showed the two groups had different behaviours (time-by-group interaction p = 0.04, Tab.2). Subsequent analyses of changes within groups showed significantly lower glucose AUC during exercise than in the corresponding period of the non-exercise day in the AER group, but not in the RES group (p = 0.04 and p = 0.90, respectively). Similar results were obtained when analyses took into account a 5-min lag-time in glucose concentration changes between blood and interstitial fluid (data not shown).

Differences between the groups were also found in the comparison of glucose AUC measured during the nocturnal period following the training session and the corresponding period of the non-exercise day (time-by-group interaction, p = 0.02, Tab.2). Again, glucose concentration AUC was significantly lower after the exercise session in the AER, but not in the RES group (p = 0.03 and p = 0.62, respectively).

**Figure 3:**
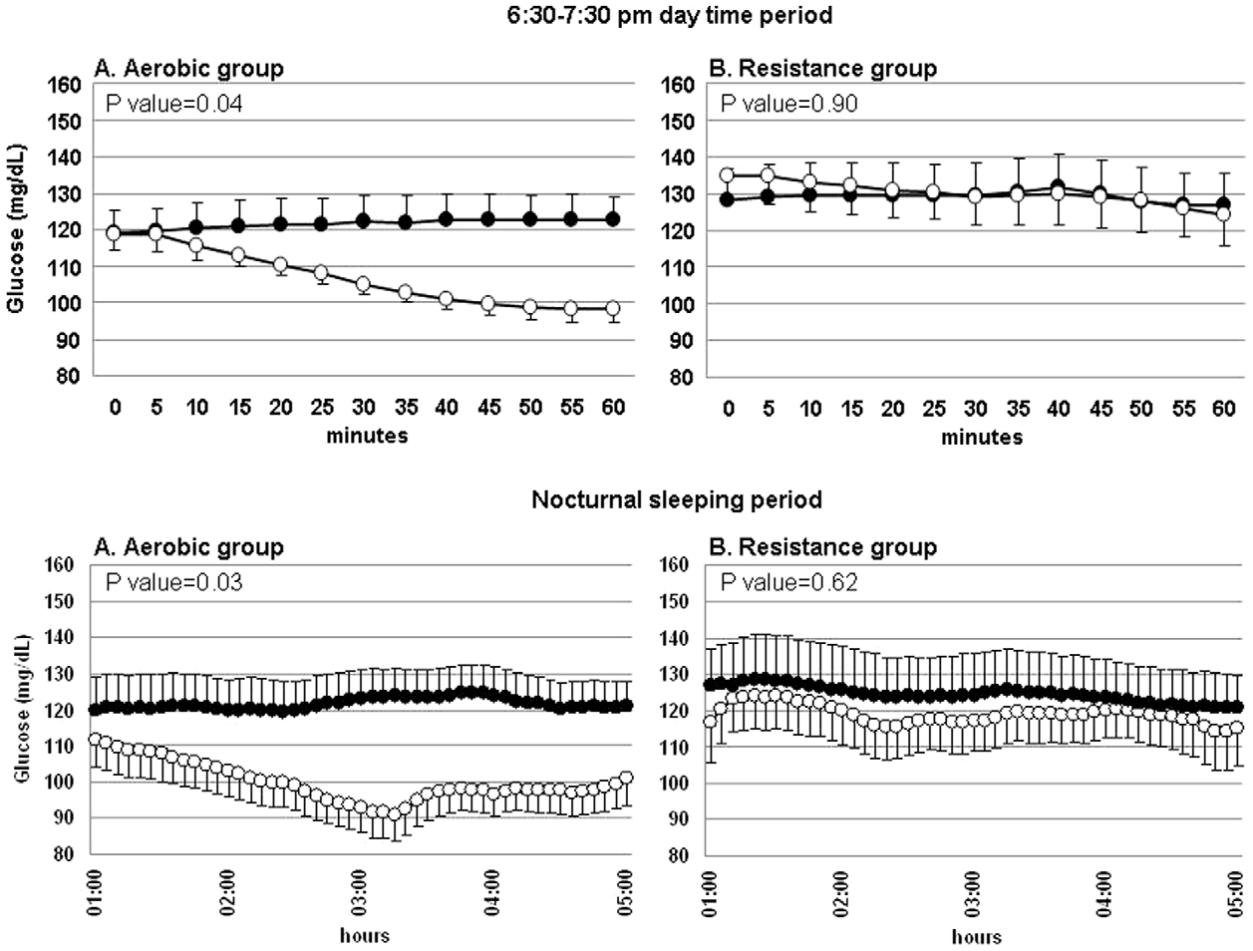
Mean glucose concentrations behavior during selected periods, in the aerobic (A) and the resistance (B) groups. Upper panels: glucose concentrations during the 60-min exercise session and the corresponding period of the non-exercise day. Lower panels: glucose concentrations during the nocturnal sleeping period (01:00–05:00 am) of the two days. White circles indicate glucose values in the exercise day, and black circles those in the non-exercise day. P values refer to differences in glucose concentration AUCs between the exercise day and the non-exercise day.

Fig. 3 shows the glucose levels behaviours in these periods, in the two groups.

No subjects had severe hypoglycaemia or symptomatic hypoglycaemias. However, CGMS recorded several asymptomatic low blood glucose readings. The LBGI, which is an index summarizing in a single figure the extent and duration of hypoglycaemias, was calculated for the nocturnal and for the 24-h periods of the two days (Tab.2). In the AER group, LBGI was significantly higher in the nocturnal period of the exercise day than in the corresponding period of the non-exercise day (p = 0.012). Similar differences were found when comparing the 24-h periods. However, this latter difference did not reach statistical significance (p = 0.08). Conversely, the RES group did not show any differences in the nocturnal or 24-h LBGI values between the two days (p = 0.88 and p = 0.34, respectively).

The HBGI, which summarizes the extent and duration of high blood glucose readings, was similar in the exercise and non-exercise days in both groups (Tab.2).

Multiple regression analysis was carried out to assess whether there were additional variables independently associated with glucose changes. However, these showed that gender, medication category (insulin sensitizers vs secretagogues), age and baseline glucose levels were not significant predictors of changes in blood glucose, either in the exercise period or in the nocturnal period or in the whole exercise day (data not shown).

HbA1c changes from baseline were assessed in these subjects at the end of the RAED2 protocol, after 4 months of intervention. HbA1c levels showed similar mean improvements in the aerobic and resistance groups (−0.48±0.14 vs −0.39±0.16%, p = 0.70).

## Discussion

In the present study we have compared the short-term effects on blood glucose concentrations of a single bout of aerobic or resistance exercise in type 2 diabetes subjects included in a regular training programme. Glucose concentrations were measured by CGMS for 48 hours, comprising a day with an exercise session and the following non-exercise day. The exercise programmes were of moderate intensity and designed according to current recommendations for physical activity for these subjects [1]. Our results showed that, although the aerobic and the resistance training induced similar long-term improvements in HbA1c levels, there were different behaviours between the groups in blood glucose readings during and for several hours after a single exercise session. In particular, there was a significant reduction of glucose concentrations during the aerobic exercise but not during the resistance exercise, and similar differences between groups were also found in the nocturnal period following the training session. Again, glucose concentrations were lower during the night subsequent to an aerobic exercise session, whereas this phenomenon did not occur after resistance exercise.

Our findings are of speculative and practical interest. Previous research showed that after a single bout of physical activity the contraction-mediated increase in muscle glucose uptake persists for several hours and the insulin mediated uptake for up to 48–72 h [1], [7], [15]. These observations suggested that the beneficial effects of exercise on long-term glycaemic control can be ascribed to the cumulative effects of the succeeding bouts of exercise. In our study, the lack of significant differences in both groups between the 24-h glucose AUCs of the exercise and non-exercise days is consistent with previous evidence of a prolonged metabolic effect of either aerobic or resistance regular training. Furthermore, the comparable 48-h glucose profiles between the two exercise groups is consistent with an analogous long-term metabolic effect of aerobic and resistance exercise, confirmed by the similar HbA1c improvement observed in these subjects after 4 months of intervention [4]. Nevertheless, the two groups showed significant differences during and for several hours after exercise. In this regard, the observed time course suggests that differences between training types in the acute glucose changes are more likely due to differences in the contraction-mediated pathway than in the insulin mediated uptake. Accordingly, improvements of insulin mediated glucose uptake were similar after 4 months of aerobic or resistance training [4]. Differences between groups in the counterregulatory hormone response to exercise may also be hypothesized. Interestingly, it was reported that exercise-induced GH secretion could be greater after resistance exercise [16]. In our study counterregulatory hormones were not assessed. Nevertheless, it seems unlikely that possible differences in secretion of these hormones might account for the differences in blood glucose between groups observed in the nocturnal period, 6–12 hours after exercise.

From a practical point of view, the recent consensus statement about exercise in type 2 diabetes, by the American College of Sport Medicine (ACSM) and the American Diabetes Association (ADA) [1], recommended combining aerobic and resistance training in these subjects, in order to lower HbA1c levels [17] and guarantee the specific benefits of both these exercise modalities [18]. However, the lack of information about possible differences in the acute effects of aerobic and resistance training on glucose concentrations did not allow the ACSM/ADA experts to suggest whether there should be any differences in medication dosage and dietary carbohydrate adjustments to physical activity in relation to the scheduled type of exercise. In this regard, it is noteworthy that our experimental design explored the effects of exercise sessions included in a training programme, to assess whether each single exercise bout causes acute changes of blood glucose over the chronic effects of a long-term training. In terms of clinical practice, this may be even more interesting than comparing an exercise day with a true non-exercise day, which is a somewhat artificial model.

Interestingly, in our study the LBGI index, which summarizes the severity and duration of hypoglycaemic episodes and is a reliable predictor of severe hypoglycaemia risk [14], increased significantly during the night following aerobic exercise, whereas it did not change after resistance exercise. It should be underlined that the risk of hypoglycaemia, as estimated by the LBGI index, was low in these diabetic patients and mildly increased after the aerobic exercise session. Nevertheless, most subjects in our study were given metformin alone, which carries a very low risk of hypoglycaemia and might even blunt the post-exercise blood glucose reduction [19], whereas a small number of them were given insulin secretagogues, i.e. received medications which substantially increase hypoglycaemia risk. Therefore, these differences in the LBGI index observed during the night after exercise, even if small, merit attention.

Some previous studies assessed the acute effects of exercise on blood glucose in type 2 diabetic subjects not involved in structured training programmes, reporting that physical activity lowered the time spent in hyperglycaemia, as compared to a non-exercise day [20]–[21]. However, to the best of our knowledge, only one recent study has compared, using a CGMS, the acute effects on blood glucose of a single session of aerobic or resistance exercise in these patients. Van Dijk et al [22] reported, in a crossover study, similar reductions in the prevalence of hyperglycaemia over a 24-h period after aerobic or resistance exercise, as compared to a non-exercise day. Moreover, in this study, a greater time spent in hypoglycaemia was reported in the evening after exercise, and this figure was somewhat higher after aerobic activity, although the study did not provide analysis of differences between the exercise types in this time frame. In our study the exercise session was undertaken in the late afternoon, when many subjects could train more easily, after work or family activities. In contrast, in the van Dijk study, exercise was carried out in the late morning. This difference may likely explain why in our study hypoglycaemia risk was higher in the nocturnal period, whereas in the van Dijk study this phenomenon occurred earlier, in the evening.

Interestingly, all recorded hypoglycaemias were asymptomatic in our subjects. It is worth noting that in subjects with long-standing type 2 diabetes, the hypoglycaemia rate is substantially higher than commonly thought, as most episodes remain undiagnosed [23], and that hypoglycaemias are considered the most likely candidate responsible for the lack of beneficial effects or even the increased risk of mortality observed during intensive glucose-lowering therapy [24]–[25].

Unlike the findings of previous studies, in our subjects the 24-h blood glucose AUC and the 24-h HBGI value, which summarizes duration and extent of hyperglycaemia, were unaffected by either aerobic or resistance exercise. Differences in the experimental design probably explain these different results. In particular, we assessed what effects a single bout of exercise has over the sustained effects of training, whereas the other studies compared exercise with a non-exercise condition.

Interestingly, findings consistent with our data were reported in a preliminary study [26] carried out in trained individuals with type 1 diabetes submitted to continuous glucose monitoring during and after aerobic or resistance exercise. Aerobic exercise caused greater reduction of blood glucose and a greater need for carbohydrate supplementation during aerobic exercise than resistance exercise. More recently, the same authors have examined in these patients the effects of combined exercise sessions, in which the aerobic training was performed either before or after the resistance training [27]. In this study, blood glucose reduction was again greater during aerobic exercise than resistance exercise, especially when the aerobic component came first. Moreover, there was a tendency to differences in AUC for post-exercise nocturnal hypoglycaemia according to the sequence in aerobic and resistance exercise, the glucose reduction being greater when aerobic exercise preceded resistance exercise.

As a whole, these data indicate that a better understanding of the acute effects of each specific type of exercise is needed, in order to maintain glucose levels in an appropriate range during and after physical activity. This is also relevant if we consider that therapeutic adjustments induced by fear of exercise-induced hypoglycaemia often cause a suboptimal metabolic control during physical activity in diabetic patients, mitigating the potential beneficial effects of exercise.

The strengths of our study are the careful experimental design and the assessment of the effects of bouts of exercise included in a regular training programme, in order to reproduce real-life conditions. To the best of our knowledge, our study is the first to assess the acute effects of exercise within a long-term training programme in type 2 diabetic subjects.

A limitation is the somewhat small sample size, although it did not prevent us from finding several statistically significant differences. Moreover, owing to the inclusion/exclusion criteria, extrapolation of the present findings to other classes of diabetic patients, such as those on insulin therapy, should be undertaken with caution.

In conclusion, although aerobic and resistance training have similar long-term metabolic effects in type 2 diabetes subjects, the acute effects of single bouts of these exercise types differ. In particular, aerobic exercise lowers blood glucose concentrations to a greater extent than resistance exercise, both during and up to twelve hours after the exercise session, carrying a higher risk of exercise-induced hypoglycaemia. Of particular concern is late-onset hypoglycaemia, especially when the exercise is scheduled in the late afternoon, as this risk occurs in the sleeping nocturnal period. These findings suggest that adjustments of medications and/or carbohydrate intake in diabetic subjects should also take into account the scheduled type of exercise. Further research is needed to establish the mechanisms underlying differences in the short-term effects of aerobic and resistance training, and to assess the acute metabolic changes induced by a combination, in the same session, of aerobic and resistance exercise.

## Supporting Information

Checklist S1 **CONSORT checklist.**
(DOC)

Protocol S1 **Trial protocol.**
(DOC)
