# Supplementary material for: Differences in the Acute Effects of Aerobic and Resistance Exercise in Subjects with Type 2 Diabetes: Results from the RAED2 Randomized Trial
Source: PLoS One. 2012 Dec 5;7(12):e49937. doi: 10.1371/journal.pone.0049937 (PMC3515569; doi:10.1371/journal.pone.0049937)
Supplement: Protocol S1 — Trial protocol. (DOC) [file pone.0049937.s002.doc]

**Protocol of the RAED2 Study, as approved by the Ethical Committee of the Azienda Ospedaliera Universitaria Integrata of Verona, Verona, Italy, and summarized on clinicaltrial.gov.**

The study will be carried out in 40 sedentary type 2 diabetic patients without diabetic complications, aged 40-70 yr, with body mass index between 24-36 kg/m2 and HbA1c between 6.5-9.0%. Admitted diabetes treatments will be diet and oral hypoglycemic agents.

Exclusion Criteria will be: moderate-severe somatic or autonomic neuropathy; coronary heart disease; peripheral or cerebral vascular disease; preproliferative or proliferative retinopathy or chronic renal failure (serum creatinine >1.4 mg/dl in females; >1.5 mg/dl in males); therapy with beta-blocker drugs; smokers; acute clinically significant intercurrent diseases; inability to perform the scheduled physical activity programs.

Subjects will be assigned, by a randomization schedule weighted by baseline BMI and fitness, to 2 groups of supervised exercise: aerobic training or resistance training. Training programs will have a similar volume and will be scheduled in three 60 min sessions per week for 4 months.

The aerobic training group will exercise on cardiovascular training equipment. The workload will be gradually increased up to 60% of the reserve heart rate. The resistance training group will perform different exercises on weight machines and free weights to exercise upper and lower extremity muscles. Participants will perform 8-10 different exercises each session, alternating abdominal, upper and lower extremity exercises. After a learning phase, in which participants will be instructed to exercise correctly with 3 series of 10-12 repetitions on each machine at 30-50% of 1RM, the workload will be gradually increased to 70-80% 1RM.

Before and at the end of the exercise training the following will be assessed: HbA1c (primary outcome), plasma glucose, lipid profile, blood pressure, insulin sensitivity (glucose clamp), beta-cell function (OGTT), body composition (Dual energy X-ray absorptiometry, DEXA), liver and muscle fat content (magnetic resonance imaging), expression in the muscle of some genes involved in ATP production, mitochondrial biogenesis and substrate utilization, markers of inflammation, oxidative stress and early vascular damage, peak oxygen uptake (VO2peak) during a maximal exercise test, other exercise tests, tissue oxygen extraction during exercise (Near-infrared spectroscopy, NIRS), energy expenditure and physical activity in daily life (metabolic Holter and questionnaire). Monitoring of blood glucose will also be carried out over a 48h period, comprising an exercise session (Continuous Glucose Monitoring System).
